# Respect the Burden

**Published:** 1875-11

**Authors:** 


### RESPECT THE BURDEN.

AN INCIDENT.

*"By the Author of John Halifax."*

Great Garibaldi, through the streets one day,
Passing triumphant, while admiring throngs,
With acclamations and exultant songs,
For the uncrowned kingly man made way—
Met one poor knave, 'neath heavy burden bowed,
Indifferent to the hero and the crowd.

His zealous followers would have driven aside,
The sorry creature; but that good man said,
Stretching a kind hand o'er the suffering head,
"Respect the burden. Then, majestic-eyed,
He paused, and passed on, no one saying him nay;
The heavy-laden also went his way.

Thou happy soul, who travelest like a king
Along the rose-strewn pathway of thy lot,
Respect the burden. Thou may'st see it, or not,
For one heart is to another a sealed thing.
Laughter there is that hideth sobs or moans;
Firm footsteps can leave blood-prints on the stones.

Respect the burden, whatsoe'er it be;
Whether loud outcries vex the startled air,
Or in dumb agony of loss, despair
Lifts her still face, so like tranquility—
Though each strained heartstring quivers, never shrinks;
"Let this cup pass from me!"—then stoops and drinks.

O heavy burden! Why 'tis borne, and how,
None knew save those who bear; and Him whose hand
Has laid it on the shoulder, and said "Stand—
Stand upright. Take this chrism upon thy brow,
My own annointed. Sore thy load may be;
But know—beneath it thou art carrying Me."

—*Good Words.*

---

Look on the Bright Side. It is the right side. The times may be hard, but it will make them no easier to wear a gloomy and sad countenance. It is the sunshine, and not cloud, that makes the flower. There is always that before and around us which should cheer and fill the heart with warmth. The sky is blue ten times where it is black once. You have troubles, it may be; so have others; none are free from them. They give sinew and tone to life, fortitude and courage to man. That would be a dull sea, and the sailor would never acquire skill, where there was nothing to disturb the surface of the ocean. It is the duty of every man to have all the enjoyment he can within him: and, above all, he should look on the bright side of things. What though things do look a little dark; the lane will turn, an d the night will end in broad day. In the long run, the great balance rights itself. What is ill becomes well; what is wrong, right. Men are not made to hang down their heads or lips, and those who do, only show that they are departing from the paths of true common sense and right. There is more virtue in one sunbeam than in a whole hemisphere of cloud and gloom. Therefore, we repeat, look on the bright side of things. Cultivate what is warm and genial—not the cold and repulsive, the dark and morose.

---

FISH FLOUR.—A novel and remarkable article of food, prepared from the products of the ocean, has lately been brought prominently forward—this is fish flour. It is not yet manufactured in any great quantity, as the article is still new in the market, and consequently there is no great demand for it. The flour is prepared in Norway, from dried cod-fish of the first quality; it is thoroughly descicated and then ground in a mill. There are two qualities, the coarse and the fine-ground. It is especially the former which has found favor with the public; from it an excellent dish of fish-balls can in a short time be prepared, while the finest ground is used for fish puddings, a dish highly appreciated in Norway and Sweden. In Catholic countries, in localities where there is no regular supply of fresh fish, it is presumed this article will be more particularly important.

---

**REFINED homes are at the end of civilization. All the work of the world—the railroading, navigating, digging, manufacturing, inventing, teaching, writing, fighting, are done, first of all, to secure each family in the possession of its own hearth; and secondly, to surround as many hearths as possible with grace, culture and beauty. The work of all races for five thousand years is represented in the difference between a wigwam and a lady's parlor It has no better result to show.**

